# MED1/337: Using the Internet to Deliver Decision Support Systems to Physicians

**DOI:** 10.2196/jmir.1.suppl1.e44

**Published:** 1999-09-19

**Authors:** MW Peterson, KW Thomas, C Dayton

**Affiliations:** ^1^University of Iowa, Iowa CityUSA

**Keywords:** Asthma, Tuberculosis, Education, Guidelines, Computer, Decision support

## Abstract

**Introduction:**

Scientifically based clinical guidelines are increasingly directing medical care. Two such guidelines are the National Asthma Education Program 2 (NAEP2) and the ATS/CDC Tuberculosis Guidelines. While these expert-derived guidelines are potentially very useful, it is difficult for individual physicians to be knowledgeable about all these guidelines and even more difficult for them to apply the guidelines to a specific patient care setting. Computer networks offer the potential to bridge this need and individualize guidelines to providers at the point of clinical contact.

**Methods:**

Using content from the NAEP2 and the TB Guidelines, we developed two computer-based delivery systems using the Internet. Using CGI and NAEP2, we developed an algorithm to stage acute asthma disease severity and deliver specific recommendations to physicians. We tested the computer-delivered algorithm using patient scenarios. Using the TB Guidelines, we also developed an html-linked decision support tool to deliver appropriate treatment recommendations based on answering three clinical questions. We again tested the utility of the program using patient scenario testing.

**Results:**

We first validated the asthma algorithm by comparing asthma expert's decisions with the decisions reached by non-pulmonary nurses using the computerized decision support system (DSS). Using the DSS, nurses scored the same as experts (89 ± 1.4% vs. 88 ± 3%; p=NS). Using the same test, we next compared internal medicine residents using the computerized DSS to residents using the paper version of NAEP2 asthma guidelines. Residents using the computerized DSS scored significantly better than residents using the paper-based NAEP2 guidelines (92 ± 1.7% vs. 84 ± 1.5%; p<.002). When we similarly compared residents using the computerized TB DSS to residents using a reference card, the residents using the computerized DSS scored significantly better (95.8% correct vs. 56.6%; p<.01).

**Discussion:**

Clinical guidelines are increasingly common in chronic disease management. Previous work has demonstrated that traditional educational interventions are minimally effective at altering physician behavior. In other settings, learning occurs best when coupled to relevant activity. Computers can aid with this learning by providing relevant information to the clinician when they need it to make clinical decisions. Using two different sets of guidelines, we have demonstrated that computer-based DSS's delivered over the Internet are more effective than traditional resources in improving physician performance as measured by compliance with guidelines. We conclude that guidelines can be adapted to deliver individualized recommendations using the Internet and that computer-delivered recommendations are effective at improving physician compliance.

